# Characterization of Transcription Factor Gene *OsDRAP1* Conferring Drought Tolerance in Rice

**DOI:** 10.3389/fpls.2018.00094

**Published:** 2018-02-01

**Authors:** Liyu Huang, Yinxiao Wang, Wensheng Wang, Xiuqin Zhao, Qiao Qin, Fan Sun, Fengyi Hu, Yan Zhao, Zichao Li, Binying Fu, Zhikang Li

**Affiliations:** ^1^National Key Facility for Crop Gene Resources and Genetic Improvement, Institute of Crop Sciences, Chinese Academy of Agricultural Sciences, Beijing, China; ^2^School of Agriculture, Yunnan University, Yunnan, China; ^3^Key Lab of Crop Heterosis and Utilization of Ministry of Education, Beijing Key Lab of Crop Genetic Improvement, China Agricultural University, Beijing, China; ^4^Shenzhen Institute for Innovative Breeding, Chinese Academy of Agricultural Sciences, Shenzhen, China

**Keywords:** DREB, drought tolerance, *OsDRAP1*, overexpression, rice, transcription factor

## Abstract

**HIGHLIGHTS**

- Overexpressing and RNA interfering *OsDRAP1* transgenic rice plants exhibited significantly improved and reduced drought tolerance, but accompanied with negative effects on development and yield.

The dehydration responsive element binding (DREBs) genes are important transcription factors which play a crucial role in plant abiotic stress tolerances. In this study, we functionally characterized a DREB2-like gene, *OsDRAP1* conferring drought tolerance (DT) in rice. *OsDRAP1*, containing many *cis*-elements in its promoter region, was expressed in all organs (mainly expressed in vascular tissues) of rice, and induced by a variety of environmental stresses and plant hormones. Overexpressing *OsDRAP1* transgenic plants exhibited significantly improved DT; while *OsDRAP1* RNA interfering plants exhibited significantly reduced DT which also accompanied with significant negative effects on development and yield. Overexpression of *OsDRAP1* has a positive impact on maintaining water balance, redox homeostasis and vascular development in transgenic rice plants under drought stress. *OsDRAP1* interacted with many genes/proteins and could activate many downstream DT related genes, including important transcription factors such as *OsCBSX3* to response drought stress, indicating the *OsDRAP1*-mediated pathways for DT involve complex genes networks. All these results provide a basis for further complete understanding of the *OsDRAP1* mediated gene networks and their related phenotypic effects.

## Introduction

Rice (*Oryza sativa*. L) is the staple food for half of the world's population. Rice production normally requires large amounts of water. Water availability and drought are becoming major constraints for rice cultivation because of greatly fluctuated and generally reduced rainfall in the majority of rice growing areas worldwide resulting from the global climate change. Rice production was dramatically affected by severe drought occurred every year in rainfed rice-growing areas worldwide (Pandey, 2009; Lenka et al., 2011). To ensure food security, it is imperative to develop drought tolerant high-yielding rice varieties.

DT is a complex trait controlled by many genes and shows significant genotype by environment interaction (Babu, 2010). Genetically, many QTLs affecting DT in rice has been identified (Li et al., 2005; Xu and Vision, 2005; Kumar et al., 2014), but few of them have been cloned and functionally characterized (Uga et al., 2013; Li et al., 2015). At the transcriptomic level, it is well known that drought can induce differential expression of large numbers of genes in rice (Wang D. et al., 2011). Of these drought responsive genes, several families of transcription factor (TF) such as AP2/ERFs, NAC, bZIPs and MYBs were found to play an important role in drought responses via regulating large numbers of downstream genes and pathways through complex transcriptional networks (Hadiarto and Tran, 2011; Todaka et al., 2015). Many TF families typically each contains many member genes, but only a portion of a TF family are functionally involved in plant responses to abiotic stresses (Joshi et al., 2016). Genes of the AP2/ERF family are characterized by a conserved AP2/ERF DNA binding domain (Riechmann and Meyerowitz, 1998; Sakuma et al., 2002). This TF family is further classified as four major subfamilies including AP2 (Apetala), RAV (related to ABI3/VP1), DREB (dehydration responsive element-binding protein), and ERF (ethylene responsive factor). There are 163 AP2/ERF genes in the rice genome (including 57 DREB members), and 70 of them were differentially regulated under different abiotic stresses (Sharoni et al., 2011). Most DREB member genes have been found to manipulate downstream stress-responsive genes by binding their drought responsive element (DRE) and GCC-box *cis*-element (Liu et al., 1998; Dubouzet et al., 2003). The DREB TFs could be further divided into DREB1 and DREB2, which were reportedly involved in two separate signal transduction pathways in plants in response to low temperature and dehydration stresses, respectively (Lata and Prasad, 2011). Four DREB1 genes (*OsDREB1A, OsDREB1B, OsDREB1C*, and *OsDREB1D*) were firstly identified in rice. Overexpression of *OsDREB1A* could enhance abiotic stress tolerance in *Arabidopsis* (Dubouzet et al., 2003) *OsDREB1F* is highly induced by drought stress and exogenous ABA application and overexpression of *OsDREB1F* is reportedly to lead to enhanced tolerance to salt, drought, and low temperature in both rice and *Arabidopsis* (Wang et al., 2008). In addition, higher expression of *OsDREB1G* in transgenic rice plants could significantly improve their tolerance to water deficit stress (Chen et al., 2008). Similarly, DREB2-type TFs are involved in a conserved regulatory mechanism in several crop plants in response to drought, salinity, and heat stresses (Lata and Prasad, 2011; Mizoi et al., 2012). There are five DREB2 genes (*OsDREB2A, OsDREB2B, OsDREB2C, OsDREB2E*, and *OsABI4*) in the rice genome (Matsukura et al., 2010; Srivastav et al., 2010). *OsDREB2A* expression in rice was evidently induced by water deficit and exogenous ABA application, which could result in improved DT (Cui et al., 2011). The transcript of *OsDREB2B* has both functional and non-functional forms. The former was markedly increased during stress conditions and was able to enhance DT by drought-induced alternative splicing of its pre-mRNA (Matsukura et al., 2010). All these results indicated that *OsDREB2s* also play important roles in plant DT.

In our breeding program, we developed a DT pyramiding line-DK151 from an F_2_ population of a cross between two IR64 introgression lines (Wang W. S. et al., 2011). In the following transcriptomic experiment using DK151 and its background parent, IR64, we found that large numbers of genes were specifically up-regulated in DK151 when compared with IR64 (Wang D. et al., 2011). Of these drought up-regulated genes in DK151, we identified a new rice DREB2-like TF gene, *OsDRAP1* (Drought Responsive AP2/EREBP gene) located in an introgressed segment on chromosome 8 of DK151 and whose expression was greatly up-regulated in DK151 by drought, suggesting its potential role in rice DT. Here, we report the functional characterization of *OsDRAP1* and evidence for its important role in improving rice DT.

## Materials and methods

### Plant materials, growth conditions, and stress treatments

Seeds of three rice genotypes, including Nipponbare, its transgenic plants overexpressing coding region of *OsDRAP1* and RNAi knock-down transgenic plants generated by *Agrobacterium*-mediated transformation, were grown under the controlled conditions in the growth chamber. For stress and hormones treatments, 2-week-old rice plants were transferred from the basal nutrient solution to nutrient solution containing 20% PEG (PEG6000), 150 mM NaCl, 100 μM ABA, 100 μM methyl jasmonate (MeJA) or 20 mM H_2_O_2_ and exposed to low temperature (4°C). Leaf tissues were then harvested as the indicated times and stored at −80°C for further analysis.

The transgenic rice plants and their background parent, Nipponbare, were evaluated for their DT performances in a pot experiment in the greenhouse of Chinese Academy of Agricultural Sciences (Beijing, China). Three healthy seedlings were transplanted equidistantly into a strip pot (50 cm in length, 15 cm in width and 15 cm in height) filled with 2 kg of sterilized field soil. Seedlings of each genotype were planted in six pots. The pots were maintained under controlled conditions with 14 h daylight at 28°C and a 10 h dark period at 25°C. In the stress treatment, drought stress was initiated at seedling and tillering stages by withholding watering at precisely determined time intervals when the soil water content reached 15, 10, and 7.5% at 1, 3, and 5 days after the drought treatment, respectively, as measured by soil moisture meters (TZS-W, Zhejiang Top Instrument Co. Ltd).

### RNA extraction, quantitative real-time PCR (qRT-PCR)

Total RNA was extracted from rice tissues of the three genotypes using TRIzol Reagent (Invitrogen, USA). cDNA synthesis was performed using EasyScript First-strand cDNA Synthesis SuperMix (TransGene, Beijing, China) according to the manufacturer's instructions. qRT-PCR analysis was carried out using TaKaRa SYBR premix Ex Taq™ according to the manufacturer's instructions. The relative expression of each gene was calculated according to the method of 2^−ΔΔCt^ (Livak and Schmittgen, 2001). Primers used for qRT-PCR were subsequently tested in a dissociation curve analysis and verified for the absence of nonspecific amplification. All analyses were performed with three biological replicates.

### Vector construction and genetic transformation

The coding region of *OsDRAP1*, was amplified from the rice cDNA by PCR using *Kpn*I and *Bam*HI linker primers. The resulting *OsDRAP1* fragment was inserted into the *Kpn*I and *Bam*HI site of pCUbi1390 (Peng et al., 2009), generating Ubipro::OsDRAP1. 275-bp *OsDRAP1* specific sequence was amplified from the rice cDNA and inserted into RNAi vector pH7GWIWG2 (II) (Karimi et al., 2005) using the Gateway technology in end-to-end orientations by using an intron as spacer. 1.8 kb-fragments of the *OsDRAP1* promoter were amplified from rice genomic DNA (Nipponbare) by PCR and inserted into the vector pMDC162 (Curtis and Grossniklaus, 2003), respectively using the Gateway technology, generating OsDRAP1-pro::GUS. All the vectors were introduced into *Agrobacterium tumefaciens* strain *EHA105*, and then transferred into Nipponbare plants via *Agrobacterium*-mediated transformation following the protocol described by Duan (Duan et al., 2012).

### Histological analyses

To determine the localization of *OsDRAP1* in rice plant tissues, the transverse sections (100 μm thick) of the OsDRAP1-pro1::GUS transgenic seedlings and roots with GUS stained were fixed for over 12 h in 2.5% glutaraldehyde buffered with 0.2 M phosphate buffer (pH 7.2). The sections were treated as described by Takemoto et al. (2002). For histological analyses, freshly collected leaves at the tillering stage were fixed immediately in FAA (3.7% formaldehyde, 5% acetic acid, 50% absolute ethanol). Plant tissues were then dehydrated, embedded, sliced, and pre-treated. Transverse section slices were ultimately stained with Haematoxylin and eosin (HE) and viewed and photographed on a microscope (DM-LS2, Leica, http://www.leica.com/).

### Physiological traits of the three genotypes under drought stress

Relative Water Content (RWC) was calculated using the following formula: RWC (%) = [(FM−DM)/(TM−DM)] × 100, where FM, DM, and TM were the fresh, dry, and turgid masses of the leaves weighed, respectively. Monitoring the fresh weight loss at the indicated time points (per hour) measured the WLRs (water loss rates) of the detached leaves (You et al., 2013). Relative electrolyte leakage (REL) or solute leakage from the sampled rice leaves were evaluated using the method of Arora et al. (1998). Percent injury arising from each treatment was calculated from the conductivity data using the equation: % injury = [(% L(t)-% L(c))/(100-% L(c))] × 100), where % L (t) and % L(c) are percent conductivity for treated and control samples, respectively. Antioxidant enzyme activity such as catalase (CAT), was determined following previously reported methods (Bonnecarrère et al., 2011). Proline and MDA concentrations of the sampled leaves were measured according to the protocol of Shukla et al. (2012).

### Subcellular localization of GFP-OsDRAP1 fusion proteins

The open reading frames (ORFs) of *OsDRAP1* were inserted into pMDC43 as C-terminal fusions to the green fluorescent protein (GFP) reporter gene driven by the CaMV 35S promoter (Curtis and Grossniklaus, 2003). For transient expression, the GFP-OsDRAP1 fusion vector constructs were transformed into *Nicotiana benthamiana* protoplasts. Protoplast isolation from tobacco leaf tissues and PEG-mediated transformation were performed according to Bart et al. (2006). Cells were incubated at 28°C in the dark overnight. The resulting green fluorescence of protoplasts expressing GFP-OsDRAP1 was observed using a confocal laser scanning microscope (LSM700, Zeiss, Jena, Germany). The 35S::GFP construct was used as a control. AHL22 was used as a nucleus marker protein (Xiao et al., 2009).

### Histochemical GUS assay

For GUS staining analysis, sample tissues or whole seedlings were submerged in the GUS staining buffer (containing 2 mM 5-bromo-4-chloro-3-indolyl glucuronide, 0.1 M sodium phosphate buffer [pH 7.0], 0.1% [v/v] Triton X-100, 1 mM potassium ferricyanide, 1 mM potassium ferrocyanide, and 10 mM EDTA), vacuum infiltrated for 10 min, and then incubated overnight at 37°C. The staining buffer was removed before the samples were cleared with 95% (v/v) ethanol and then observed using a stereoscope (LEICA, 10447157, Germany).

### Transactivation analysis in yeast

*OsDRAP1* in transgenic plants was examined for the presence of one of its activation domains using a yeast assay system as described (Liu et al., 2013). A series of coding region fragments of *OsDRAP1* were amplified by PCR using *Nco*I and *Pst*I linker primers. The resultant PCR products were digested with *Nco*I and *Pst*I and then cloned into the *Nco*I and *Pst*I sites of pGBKT7, generating BD-OsDRAP1, BD-OsDRAP1ΔA, BD-OsDRAP1ΔAB, BD-OsDRAP1ΔD, BD-OsDRAP1ΔCD, BD-OsDRAP1ΔAD. An empty vector (BD) was used as a negative control. The constructs and the negative control BD were transformed into *AH109*, respectively according to the protocol of the manufacturer in the Leu-medium. About 2 days, the positive transformants verified by PCR were dropped on Leu- and Leu-His-Ade- medium, respectively. The transcriptional activation activities were evaluated according to their growth status.

### Transient transcription dual-luciferase (dual-LUC) assays

A previously described dual-luc method using *Nicotiana benthamiana* plants (Liu et al., 2008) was used for transient transcription assays. The effector plasmids, pMDC43-OsDRAP1 and pMDC43-OsDRAP1ΔD were constructed as described above. The reporter plasmid, pGreen-GCC/DRE-LUC, encodes two luciferases, the firefly luciferase controlled by the recombinant GCC box promoter or DRE *cis*-element, and the Renilla (REN) luciferase controlled by the constitutive 35S promoter. The recombinant GCC box and DRE *cis*-element promoters contain two wild-type GCC box (ATAAGAGCCGCCACTCATAAGAGCCGCCACT) and DRE *cis*-element (ATACTACCGACATGAGATACTACCGACATGAG), respectively. Their mutant GCC box (mGCC, ATAAGATCCTCCACTCATAAGATCCTCCACT) and DRE *cis*-element (mDRE, ATACTACTGATATGAGATACTACTGATATGAG) fused to the minimum 35S promoter, were PCR-amplified from the 35S template and cloned into the *Apa*I and *Sac*II sites of the vector pGreen-0800-LUC, then transformed into *Agrobacterium* (strain EHA105) containing the helper plasmid, pSoup-P19, that also encodes a repressor of co-suppression. The *Agrobacterium* strain containing both the reporter pGreen-GCC/DRE-LUC and helper pSoup-P19 was used alone or mixed with the *Agrobacterium* strain containing the effector plasmids pMDC43-OsDRAP1 or pMDC43-OsDRAP1ΔD.

The reporter strain was either incubated alone or incubated as a mixture with the effector strain (at the reporter: effector ratio of 1:9 or 2:8). *Agrobacteria* suspension in a 10 ml syringe was carefully press-infiltrated manually onto healthy leaves of *Nicotiana benthamiana*. Plants were left for 3 days after infiltration. Leaf samples were collected for the dual-luc assay using commercial Dual-LUC reaction (DLR) reagents, according to the manufacturer's instruction (Promega). After measurement of the firefly luciferase activity, 40 ul of the Stop and Glow buffer (Promega) was added to the reaction to quench the firefly luciferase and to initiate the REN luciferase (REN) reaction.

### Yeast two-hybrid assay

The protein interaction analysis was performed using Matchmaker Two-Hybrid System 3 (Clontech, http://www.clontech.com/). A bait gene is expressed as a fusion to the GAL4 DNA-binding domain (DNA-BD), while cDNA is expressed as a fusion to the GAL4 activation domain-AD (Fields and Song, 1989; Chien et al., 1991). Construct pGBKT7-OsDRAP1ΔD above was used as a bait protein. An AD fusion library of rice leaves under drought condition (pGADT7-library) was constructed in Oebiotech (Shanghai). pGBKT7-OsDRAP1ΔD and pGADT7-library plasmids were co-transformed into *AH109*, and then were spread on SD/–Ade/–His/–Leu/–Trp/X-α-gal plates. These plates were incubated at 30°C until colonies appear. To identify the gene responsible for a positive two-hybrid interaction, we rescue the gene by PCR colony screening. After that, we sequenced the cDNA inserts and blast the sequences in NCBI databases. Finally, we retest the interaction by cotransformation into the *AH109*. In the same way, cotransformation mixtures were spread on SD/–Ade/–His/–Leu/–Trp/X-α-gal plates. These plates were incubated at 30°C until colonies appear.

### Bimolecular fluorescence complementation (BiFC) assay

Bimolecular fluorescence complementation (BiFC) assay analyses were performed as previously described (Sparkes et al., 2006). Complementary DNAs of OsDRAP1 and OsCBSX3 were cloned into BiFC vectors pnYFP-X and pcCFP-X, respectively. Pair of constructs was co-transformed into the leaves of 3-week-old tobacco (*Nicotiana benthamiana*) by *A. tumefaciens* infiltration as the above protocol in dual-LUC assays. Cells co-transformed with pnYFP-OsDRAP1/pcCFP-GUS, pnYFP-GUS/pcCFP-OsCBSX3, were used as negative controls. AHL22-mRFP was used as a nuclear localization marker protein (Xiao et al., 2009).

### Protein co-immunoprecipitation (Co-IP) assay

Recombinant constructs GFP-OsCBSX3 and Myc-OsDRAP1 were introduced into tobacco leaves as the above protocol in dual-LUC or BiFC assays, respectively, and protein extracts were prepared as described by Sawa et al. (2007). The protein extracts were precipitated with anti-Flag agarose beads (Abmart, http://www.ab-mart.com/) or anti-Myc agarose beads (CMC Scientific, http://www.cmcscientific.com) overnight. Then proteins bound to beads were resolved by SDS-PAGE and detected with Western blot using anti-GFP antibody (Sigma), and anti-Myc antibody (MBL, http://www.mblintl.com/).

### RNA-seq analysis

Three top leaves for each sample (two replicates for each sample) were harvested for each genotype under 1 day and 3 days of drought stress and control conditions. The RNA-seq sequencing and assembly were performed by Beijing CapitalBio Corporation as described in previous study (Huang et al., 2014). The number of mapped clean reads for each gene was counted and normalized into the reads per kilo base per million value (Mortazavi et al., 2008); Cuffdiff (Trapnell et al., 2009) was then used to identify DEGs. Finally, genes with a *p* ≤ 0.001 were designated as significantly differential expressed between each pair of samples. Gene function annotations were performed based on the Rice Genome Annotation Project version 7 (Kawahara et al., 2013). AgriGO was used to perform GO enrichment analysis (Du et al., 2010). The raw RNA-seq data are available in the Genome Sequence Archive in BIG Data Center (Beijing Institute of Genomics, Chinese Academy of Sciences) under the accession number PRJCA000683.

## Results

### Identification and characterization of *OsDRAP1*

In our previous transcriptomic analyses (Wang D. et al., 2011), we identified a set of AP2/EREBP TF genes that were greatly up-regulated in leaves and panicles in drought tolerant pyramiding line DK151 under drought stress. One of these AP2/EREBP TF genes, designated as *OsDRAP1* (LOC_Os08g31580), was selected for further function confirmation in this study.

**Figure 1:**
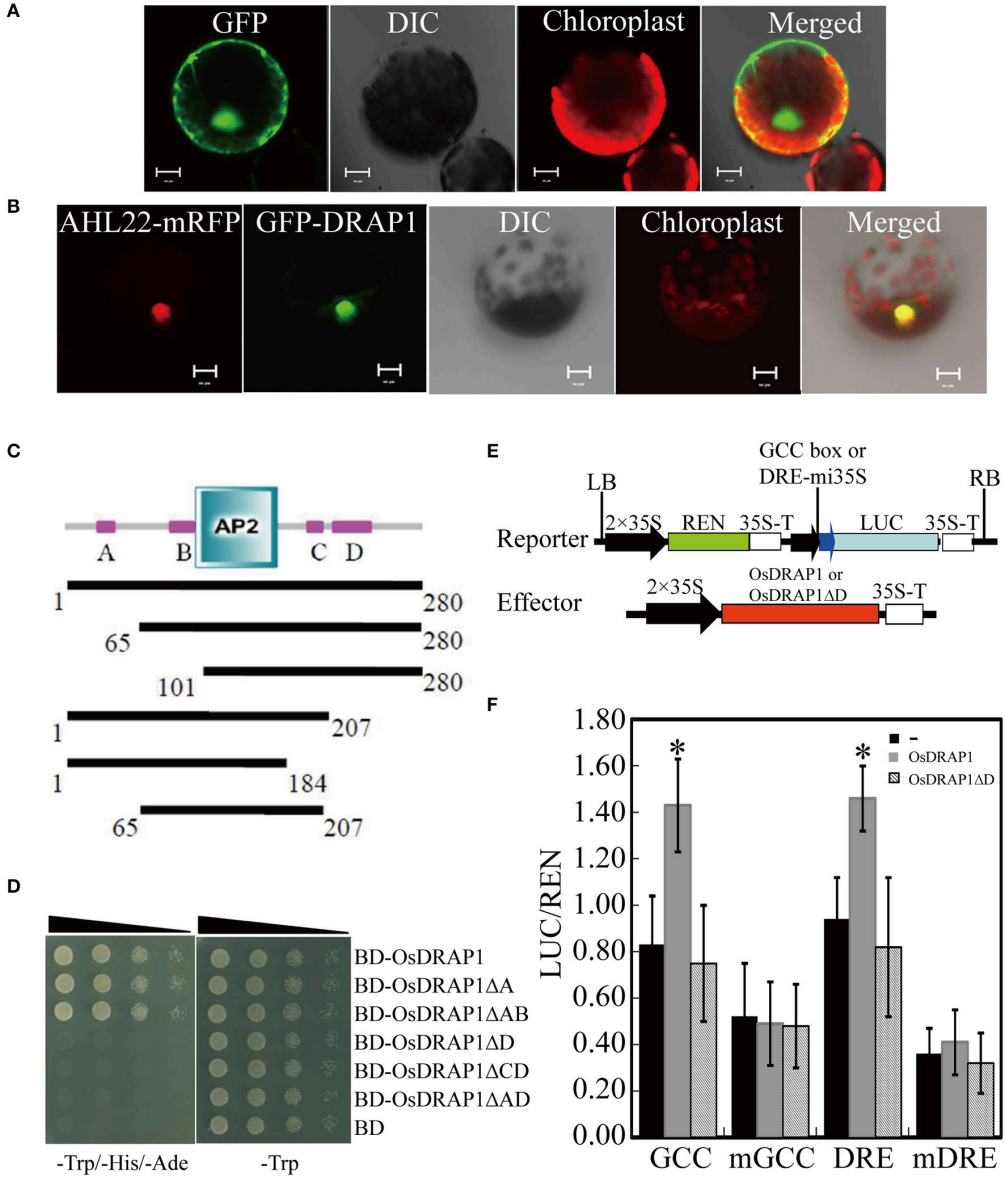
OsDRAP1 is a typical transcription activator. **(A)** GFP extensively localized in both nucleus andcytoplasm in the *N. benthamiana* protoplast; **(B)** GFP-*OsDRAP1* positively localized in the nucleus, in which AHL22 used as the nucleus marker protein, DIC indicating its differential interference contrast transmission, chlorophyll fluorescence in red and the merged image, with the scale bars in 5 μm. **(C)** The structure of the *OsDRAP1* protein and analysis of its transcription activating domains in yeast; Domains of the *OsDRAP1* protein and different lengths of bait proteins with different domain deletions; **A–D** indicate four unknown domains. **(D)** Transcription activating domain identification of *OsDRAP1* in yeast in which Yeast suspension was diluted to 10^−1^, 10^−2^, 10^−3^ times and dripped to the SD (-Trp/-His/-Ade) and SD (-Trp) medium, with pGKBT7 (BD) used as the negative control. **(E)** The schematic diagram of the reporters and effectors; **(F)** The transcription activation assay of *OsDRAP1* and *OsDRAP1*ΔD in *N. benthamiana*, with “-,” *OsDRAP1* and *OsDRAP1*ΔD indicating no effector control, full length and D domain deletion of the *OsDRAP1* proteins, respectively, with the asterisks indicating significant differences at *p* < 0.05 in *t*-tests (vs. no effector control, *n* > 10).

The *OsDRAP1* gene sequence has a full-length of 843 bp without any intron and encodes a polypeptide of 280 amino acids, consisting of an AP2 domain (103–166 aa) and four putative unknown domains (A: 25–38 aa, B: 82–100 aa, C: 190–201 aa, D: 210–239 aa; Figure 1C). The phylogenetic analysis indicated that *OsDRAP1* was closely related to members of the DREB2 subfamily in rice and wheat (Figure S1; Sharoni et al., 2011). *OsDRAP1* contains several stress- or hormone-responsive *cis*-elements in its promoter region (2.0 Kb upstream of the start codon), including 5 MYB binding sites (MBS), 6 ABA-responsive elements (ABRE), 2 gibberellin-responsive elements (GARE), and a CGTCA-motif for the MeJA-responsiveness (Table 1), implying that the expression of *OsDRAP1* could be regulated by phytohormones. Our results from cytological analyses (Figures 1A,B) indicated that *OsDRAP1* was localized in the nucleus and functioned as a nuclear protein, with its transactivation activity located primarily in region D (207-280 aa) (Figures 1C,D).

**Table 1:** *Cis*-elements analysis of the *OsDRAP1* promoter sequence.

***Cis*-Element**	**Number**	**Function**
ABRE	6	*cis*-acting element involved in the abscisic acid responsiveness
MBS	5	MYB binding site involved in drought-inducibility
CGTCA-motif	1	*cis*-acting regulatory element involved in the MeJA-responsiveness
GARE-motif	2	gibberellin-responsive element
TC-rich repeats	1	*cis*-acting element involved in the defense and stress responsiveness
motif I	1	*cis*-acting regulatory element root specific

To determine if *OsDRAP1* is involved in the activation of multiple stress signaling pathways by interacting with GCC box or DRE element (Hao et al., 1998; Cheng et al., 2013), we performed a dual luciferase reporter assay by constructing reporter vectors containing GCC box mi35S-LUC, DRE mi35S-LUC, mutated GCC- and DRE-LUC (mGCC box mi35S-LUC, mDRE mi35S-LUC), and effector vectors containing 35S::*OsDRAP1* and 35S::*OsDRAP1*Δ*D*. The results (Figures 1E,F) showed that the full length of *OsDRAP1* could significantly enhance the LUC activity in the reporters of the GCC box mi35S-LUC and DRE mi35S-LUC, but had no effect on the two mutated reporters. The effector containing the truncated *OsDRAP1* with deletion of region D (*35S::OsDRAP1*Δ*D*) could not activate all reporters. All these results indicated that *OsDRAP1* was able to activate the expression of downstream genes by binding *cis*-elements of the GCC box and DRE.

### The spatial-temporal pattern of *OsDRAP1* and its responses to different abiotic stresses and plant hormones

**Figure 2:**
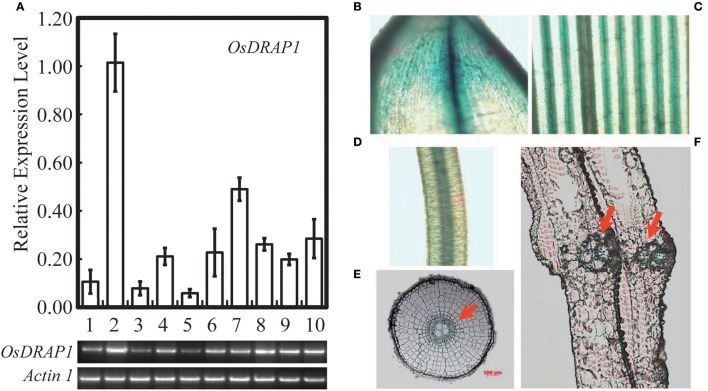
Expression model of *OsDRAP1* in different tissues of rice, in which **(A)** the transcript levels were determined by qRT-PCR and semi RT-PCR, *OsDRAP1* transcript levels in young panicles (1), matured leaves (2), leaf sheaths (3), nodes (4), internodes (5), stem bases (6), matured roots (7), young leaves (8), young roots (9) and callus (10) with *Actin1* used as the reference gene. The *OsDRAP1* expression in different tissues of the OsDRAP1-Pro::GUS transgenic rice plants by GUS staining analysis, in the shell **(B)**, leaf blade **(C)**, root **(D)**, root cross section **(E)** and sheath cross section **(F)** with red arrows indicating the vascular bundles (the scale bars are in 100 μm).

**Figure 3:**
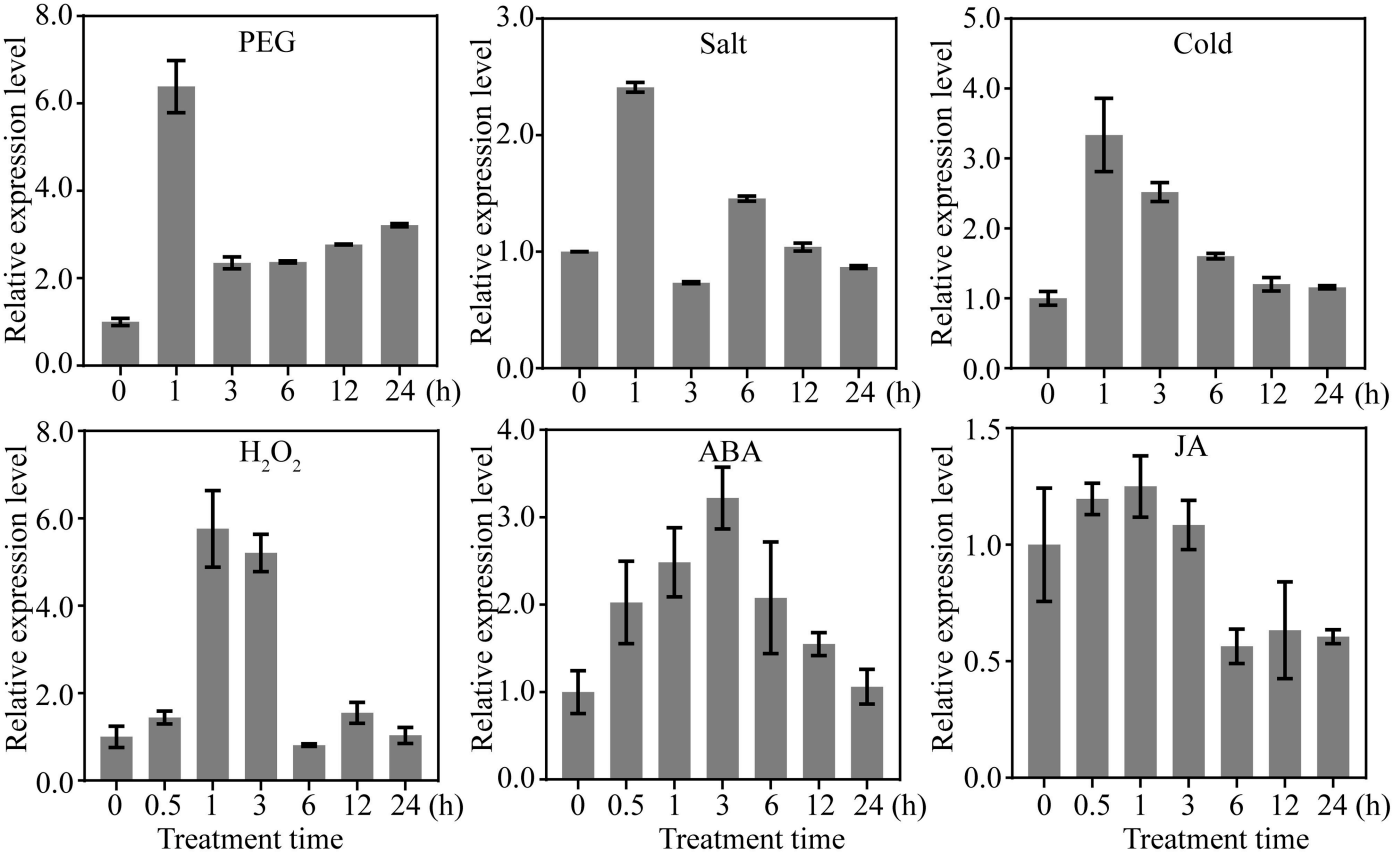
Expression profiles of *OsDRAP1* in response to various abiotic stresses and exogenous hormones; in which 14-day-old seedlings of *Nipponbare* treated with PEG (20%), salt (150 mM), cold (4 C°), 100 mμ MeJA, 100 μM ABA and 20 mM H_2_O_2_, with *Actin 1* used as the endogenous control.

Because *OsDRAP1* was differentially expressed under drought stress in a spatial-temporal manner (Wang D. et al., 2011), we examined its expression patterns in different tissues at different developmental stages using qRT-PCR. The results showed that *OsDRAP1* was highly expressed in the mature leaves and roots, but had relatively lower expression in young panicles, leaf sheaths and internodes (Figure 2A). The expression of *OsDRAP1* was induced by PEG-simulated drought, high salt, low temperature, H_2_O_2_, ABA (Figure 3). Generally, *OsDRAP1* showed similarly high expression in response to drought, salt and cold with the peak expression at 1 h after the treatments and maintaining a relatively high level of expression afterwards. Of the three plant hormones, *OsDRAP1* responded much more strongly to ABA and H_2_O_2_ than to JA, though it showed the highest expression at 1 or 3 h after the treatments and decline afterwards in the H_2_O_2_ and ABA treatments. All these results indicated that the *OsDRAP1* expression is induced by abiotic stresses, primarily by drought and salt, in a more H_2_O_2_ and/or ABA dependent manner.

### Enhanced DT and phenotypic effects by overexpression of *OsDRAP1* in rice

**Figure 4:**
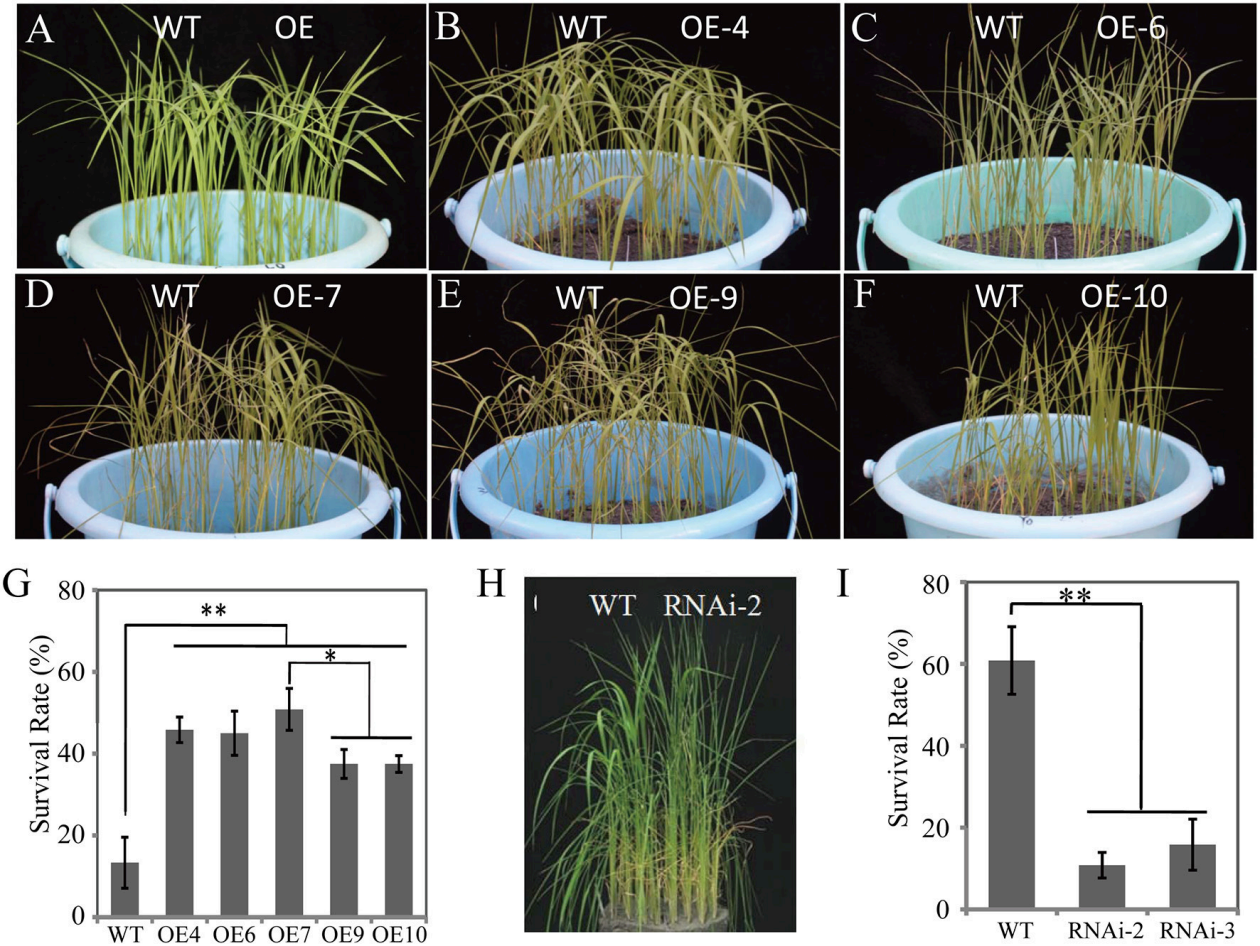
The seedling phenotypes of *OsDRAP1* OE and RNAi lines at the seedling stage under the drought stress treatments: before drought **(A)** and 5 days after drought **(B–F,H)**, and the seedling survival rates **(G,I)** evaluated 7 days after re-watering, in which the error bars indicate SD based on data of 3 replicates and the asterisks (^*^ or ^**^) indicate the significant differences at *p* < 0.05 or *p* < 0.01 in *t*-tests when compared with WT (*n* > 100).

To determine the biological function(s) of *OsDRAP1* in response to abiotic stresses, we constructed overexpressing and RNAi knockdown vectors including UbiPro::*OsDRAP1* and *OsDRAP1*-RNAi (see details in Materials and Methods). Thirteen *OsDRAP1*-overexpressing lines (OE-1 to 13) and four RNAi lines (RNAi-1 to 4) were obtained. The qRT-PCR results showed that the expression of *OsDRAP1* was up-regulated in OE lines, but down-regulated in RNAi lines to various extents when compared with WT under the normal growth conditions (Figure S2). The seedlings of the OE lines and WT plants showed no evident differences before the stress treatments and 5 d after drought (Figures 4A–F), but all transgenic OE lines exhibited improved DT compared to WT with significantly higher seedling survival rates 7 days after rewatering (Figure 4G). As expected, the two RNAi lines were more sensitive to drought stress with significantly decreased survival rates compared to WT (Figures 4H,I). These results showed that the expression of *OsDRAP1* was positively involved in DT. Based on these results, the transgenic lines, OE-7 and RNAi-2 were selected for further characterizing the functionality of *OsDRAP1* in the following experiments.

**Figure 5:**
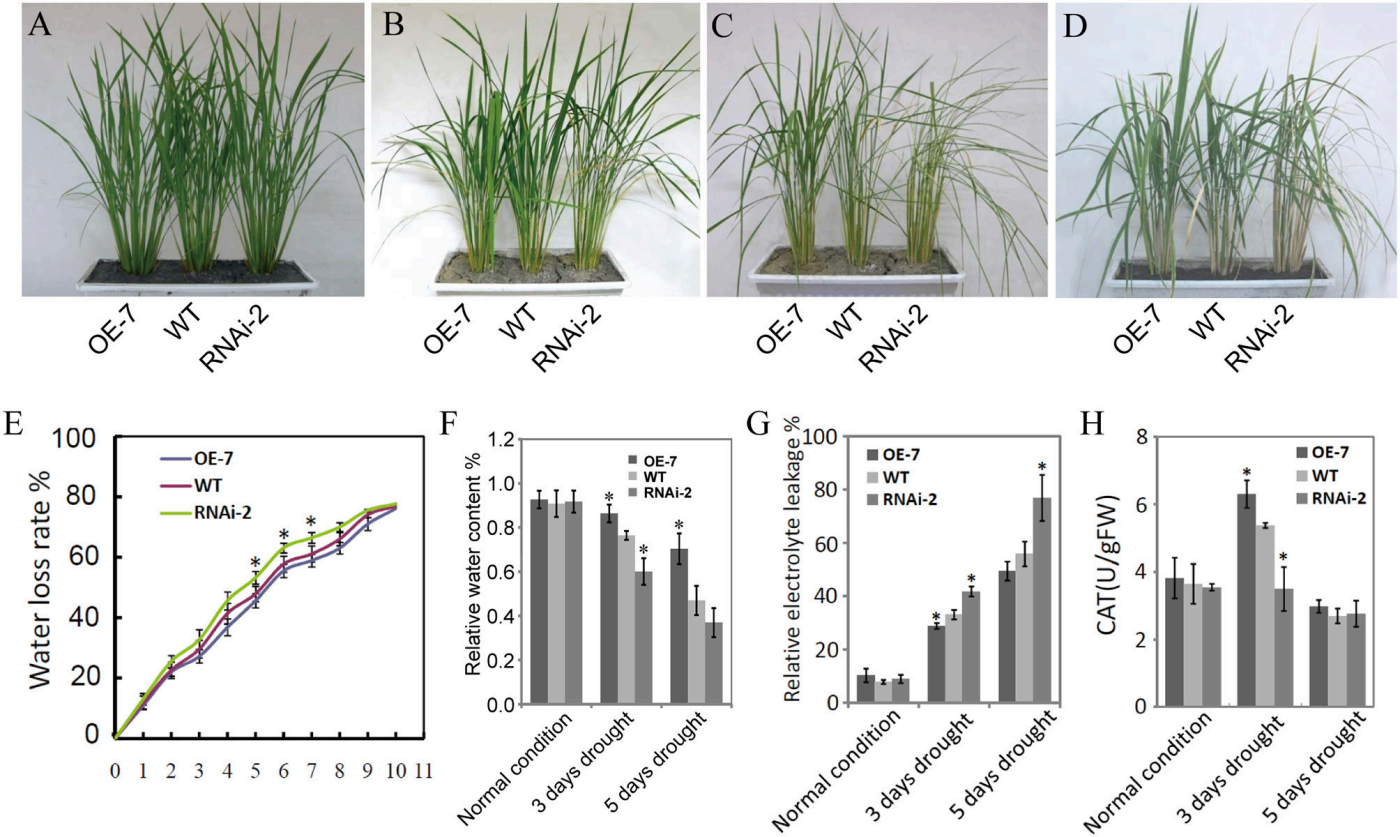
Phenotypic and physiological trait performances of the *OsDRAP1* overexpression line (OE-7), RNAi line (RNAi-2) and wild type under drought stress; The seedling growth phenotypes of OE-7, RNAi-2 and WT under the normal irrigation **(A)**, 3d and 5d drought stress **(B,C)**, and 5d after re-watering **(D)**; **(E)** The assay of water loss rates of OE-7, WT and RNAi-2; **(F)** The relative leaf water content of OE-7, WT and RNAi-3 under normal control, 3d and 5d drought stress; **(G)** Detection of relative electrolyte leakage of OE-7, WT and RNAi-2 under control, 3d and 5d drought stress conditions; **(H)** Detection of activities of ROS-scavenging enzymes (CAT) in three rice genotypes subjected to drought stress, with each column representing mean ± SD (three replicates) and ^*^ indicating a significant difference at *p* < 0.05 vs. WT based on the Dunnett's multiple comparison tests in ANOVA.

Figure 5 shows the results on the evaluation of the *OsDRAP1* overexpression line (OE-7), RNAi line (RNAi-2) and WT at the tillering stage for phenotypic and physiological trait performances under 3 and 5 days drought stress. There were no significant differences among three lines under the normal growth conditions. However, the WT and RNAi-2 plants started to show leaf rolling and wilting 3 d after drought stress, while the OE-7 was more viable than RNAi-2 and WT 5 days after drought stress and showed much better recovery 5 days after rewatering from the drought stress damage when the RNAi-2 plant was still wilted (Figure 5D). Furthermore, the OE-7 showed the lowest water loss, highest relative leaf water content, the lowest relative electrolyte leakage, and highest activities of ROS-scavenging enzymes during drought stress, while the opposite was true for the RNAi-2 plant (Figures 5E–H). All these results strongly suggested that *OsDRAP1* overexpression improved drought tolerance in rice by maintaining higher leaf water content and enhancing activities of ROS-scavenging enzymes. However, at the maturity, the OE-7 and RNAi-2 plants showed significantly reduced height by 6 cm and 16 cm when compared with the WT plant (Figure S3). In addition, when compared to the WT plant, the OE-7 plant showed significantly reduced spikelet fertility while the RNAi-2 plant showed significantly reduced panicle numbers/plant (Figures S3C,E). As a result, both the OE-7 and RNAi-2 plants had lower grain yield/plant than the WT plant (Figure S3F). All these results indicated that overexpression or repression of *OsDRAP1* had negative effects on the development and grain yield of rice, though the latter's effect was more severe.

**Figure 6:**
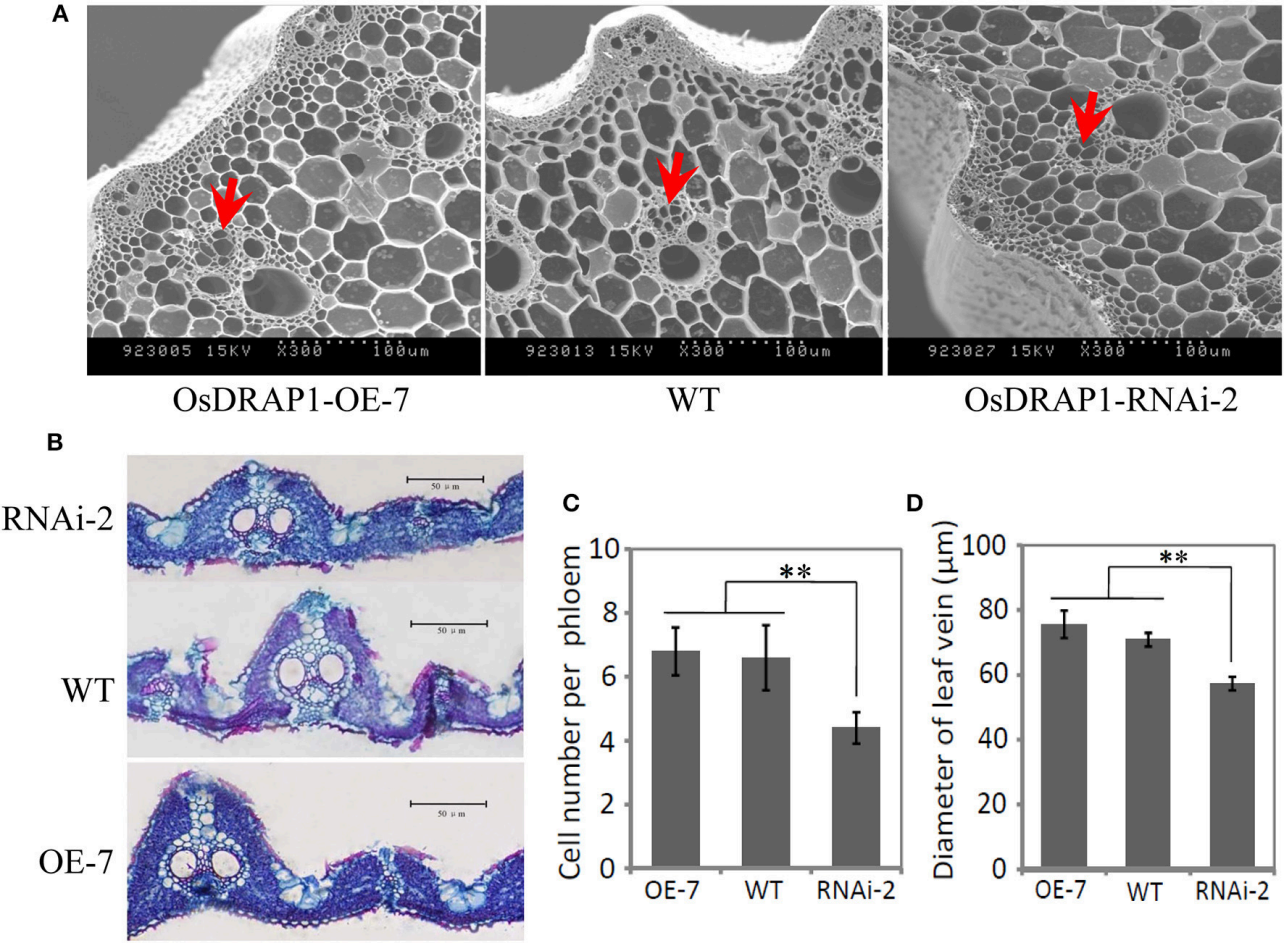
The cellular observation of the vascular tissues in one overexpression line (OE-7), one RNAi line (RNAi-2) and non-transgenic wild type (WT) for *OsDRAP1*, **(A);** and the cross section of the stem and leaf blade **(B)** at the tillering stage. Red arrows indicate the vascular bundles (phloem), with scale bars in 100 μm; **(C)** The mean cell number per phloem in the vascular bundle of the OE-7, WT and RNAi-2 plants; and **(D)** The diameter of leaf veins of the OE-7, WT and RNAi-2 plants, with the asterisks (^**^) indicating significant differences at *p* < 0.01 in *t*-tests when compared with WT (*n* > 10).

GUS staining results of the OsDRAP1-Pro::GUS transgenic plants showed that OsDRAP1 was mainly expressed in rice vascular tissues, especially in the vascular bundle of glume, leaf vein, pericycle and sheaths (Figures 2B–F). Electron microscope scanning of the stem cross sections revealed significant differences in cell number and diameter of vascular bundle between of the RNAi-2 and WT or OE-7 rice plants. Specifically, the RNAi-2 plant had fewer vascular cells and smaller diameter of vascular bundle than the OE-7 and WT plants (Figure 6), suggesting that *OsDRAP1* may play an important role in vascular development.

### Downstream genes and pathways involved in DT regulated by *OsDRAP1*

To gain insights into genes and pathways for DT regulated by *OsDRAP1*, we performed transcriptome sequencing to screen the differentially expressed genes in OE-7 compared to WT plants under normal growth and drought conditions, respectively. Table S1 shows 42 and 46 genes that were up- and down-regulated in OE-7, respectively, when compared to WT under the normal growth conditions. GO analysis revealed that the majority of these genes were functionally related to “response to stress” and “metabolic process.” After analyzing the *cis*-elements in the promoters of the 42 up-regulated genes in OE-7 using PLACE, we found that many of them have the binding sites of DREB TFs, GCC box and DRE *cis*-elements (Table S1), including GCCCOREC (S000430), DRECOREZMRAB17 (S000402) and DRECRTCOREAT (S000418), indicating that these genes were most likely directly regulated by *OsDRAP1* and function in pathway(s) contributing in the basal DT of rice.

Under drought stress, there were 85 and 53 genes up- and down-regulated in OE-7 as compared to WT (Table S2). Most of the up-regulated genes in OE-7 were functionally categorized in metabolic processes (19), response to stress (14) and transcription regulation (6). Several TFs (*OsEATB, OsERF5, OsMYB4*, and *OsWRKY89*) were evidently induced in OE-7 by drought stress. In addition, a gene encoding cellulose synthase-like family A (CSLA11) reportedly involved in cell wall biogenesis (Wang et al., 2010), was remarkably up-regulated in OE-7. Meanwhile, a set of genes related to the JA signaling pathway such as *OsJAZ8* (Yamada et al., 2012), *OsJAZ13* (Singh et al., 2015), *Jasmonate-zim-domain protein 1*, and *Jasmonate O-methyltransferase* were simultaneously induced in OE-7 under drought, indicating that JA was also involved in the *OsDRAP1* mediated regulating pathways of DT.

### Identification of the interacting proteins of OsDRAP1

**Figure 7:**
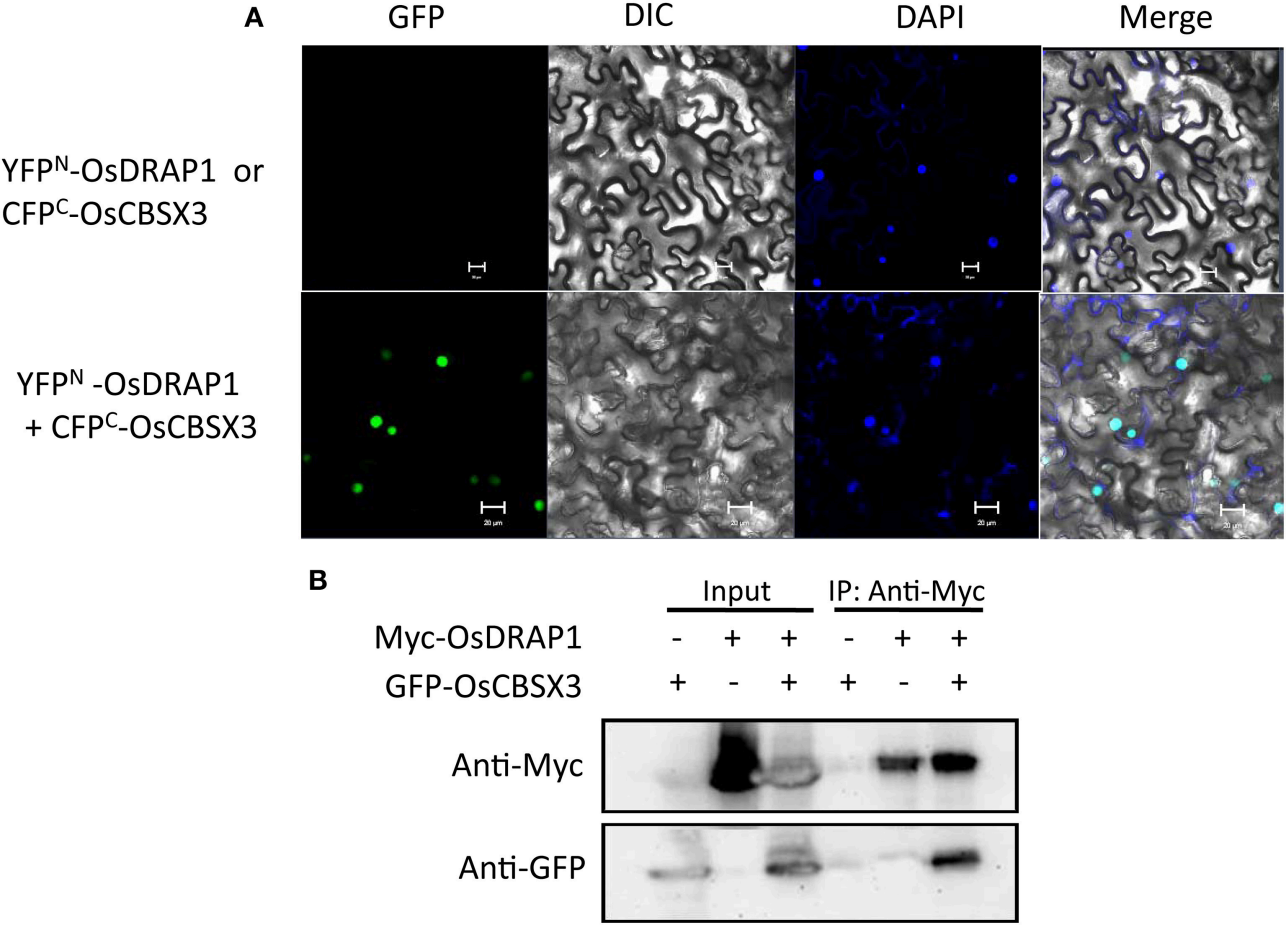
Evidence of the interaction of OsDRAP1 with OsCBSX3. **(A)** the BiFC assay showing the interaction of OsDRAP1 with OsCBSX3 in the nuclei of *N. benthamiana* leaf cells with YFP^*N*^-OsDRAP1 or CFP^*C*^-OsCBSX3 co-transformed withCFP^C^-GUS orYFP^N^-GUSserved as the negative control and scale bars in 10 μm; **(B)** the Coimmunoprecipitation assays of OsDRAP1 and OsCBSX3 with GFP-OsCBSX3 or Myc-OsDRAP1 used as the negative control.

To identify proteins that directly interact with OsDRAP1, we performed yeast two-hybrid (Y2H) screening, which identified 30 interacting proteins from 45 positive clones screened according to their α-galactosidase activity (Table S3). Genes encoding these interacting proteins were predominant in function categories of response to stress (9), metabolic process (8) and nucleotide binding (7). These genes included several previously reported genes involved in response to abiotic stresses, such as *OsRZFP34* for stomata opening, *OsAPX2* and *OsCATC* involved in redox homeostasis under abiotic stress, *OsRACK1A* in ABA and H_2_O_2_ signaling pathways and *OsCBSX3* involved in biotic stress tolerance (Lin et al., 2012; Zhang et al., 2013, 2014; Hsu et al., 2014; Mou et al., 2015). Results from bimolecular fluorescence complementation and co-immunoprecipitation assays revealed the direct interaction between A CBS (cystathionine beta-synthase) domain containing protein (OsCBSX3) and OsDRAP1 in the nucleus (Figure 7), indicating that *OsCBSX3* is involved in the *OsDRAP1*-regulated pathway in rice.

## Discussion

In plants, many DREB1 genes are known to be responsive to abiotic stresses and have been characterized, but only a few DREB2 genes have been functionally elucidated (Lata and Prasad, 2011). In this study, we have demonstrated that *OsDRAP1* in rice encodes a DREB2-like protein and functioned, as one of the DREB proteins in the AP2/ERF sub-family and plays an important role involved in several signal transduction pathways in response to different abiotic stresses, including drought, salt, cold and H_2_O_2_ stresses. *OsDRAP1* was previously characterized as a drought stress-induced AP2/EREBP gene (Wang D. et al., 2011). In this study, *OsDRAP1* was proved as a nuclear-localized protein and possessed transactivation activity which involved in ABA and JA transcriptional regulation, and *OsDRAP1* could activate the transcription of downstream genes by binding GCC box and DRE elements. The unique spatial expression pattern of *OsDRAP1* in vascular tissues and its effects causing reduced cell number and diameter of vascular bundle in RNAi plants suggest that *OsDRAP1* is involved in vascular bundle development. Although the vascular tissue is responsible the transport of water and nutrients in plants (Campbell and Turner, 2017), how vascular tissue responds to drought stress remains to be elucidated at the molecular level.

The presence of many different functional motifs in the promoter region of *OsDRAP1* suggests that *OsDRAP1* functions by mediating many downstream genes and pathways. Indeed, we were able to detect many genes functioning in response to stress and metabolic process were differentially expressed in the *OsDRAP1* OE lines, and most of these genes have GCC box and DRE elements in their promoters regulated by *OsDRAP1* (Cheng et al., 2013). Those genes also included a few TF genes such as *OsEATB, OsERF5, OsMYB4* and *OsWRKY89* that were previously reported to be involved in plant development or abiotic stress tolerance (Vannini et al., 2007; Wang et al., 2007; Zhou et al., 2010; Qi et al., 2011). Clearly, the *OsDRAP1* mediated DT genetic pathways underlying abiotic stress tolerances are involved in complex synergistic relationships with these TFs. Other important DT genes downstream of *OsDRAP1* included *CSLA11* that encodes a cellulose synthase-like family A protein. The CSLA gene family is reported to be involved in cell wall biogenesis (Wang et al., 2010; Liepman and Cavalier, 2012) and increased expression of *CSLA11* which might explain the strong effect of *OsDRAP1* on vascular development. We noted that several other genes the JA signaling pathway were strongly activated by *OsDRAP1* in the OE line, including *OsJAZ8, OsJAZ13*, Jasmonate-zim-domain protein 1 (LOC_Os03g08330) and Jasmonate O-methyltransferase (LOC_Os06g21820), implying that JA was involved in coordinate regulation of downstream genes in the *OsDRAP1* mediated drought stress responses, as reported previously (Ahmad et al., 2016). Other genes interacting with OsDRAP1 included OsCBSX3/OsBi1 (LOC_Os02g57280) and a CBS (cystathionine beta-synthase) domain containing protein (CDCPs). *OsCBSX3/OsBi1* was previously reported to be induced in rice by herbivore feeding and water deficit stress (Wang et al., 2004) and have a positive regulating role in rice resistance to *M. oryzae* in JA-mediated signaling pathways (Mou et al., 2015). CDCPs were reported to play an important role in plant responses to abiotic stresses by regulating many enzymes and maintaining the intracellular redox balance (Kushwaha et al., 2009; Yoo et al., 2011; Singh et al., 2012). OsCBSX3 might interact with OsDRAP1 and function positively in DT by maintaining the redox homeostasis under drought stress, however, the detailed molecular mechanisms how *OsCBSX3* is involved in the *OsDRAP1*-mediated DT genetic pathways remains to be elucidated.

Moreover, our results indicated that like many other plant DREB proteins, overexpression of *OsDRAP1* enhanced rice tolerance to drought and salinity, while knockdown of *OsDRAP1* lead to more sensitivity to drought in addition to significant negative effects on rice development and grain yield, as reported previously in overexpression experiments of other plant DREB genes (Kasuga et al., 1999, 2004; Ito et al., 2006). Clearly, this kind of fitness costs was much more severe in the *OsDRAP1*-knockdown plants than its constitutive overexpression-plants. This suggests it may be difficult to improve rice DT or salt tolerance by transgenic overexpression of *OsDRAP1*. Therefore, efficient utilization of *OsDRAP1* for improving rice tolerance to drought and salt requires more complete understanding of the *OsDRAP1* mediated gene networks and pathways and their related phenotypic effects.

## Conclusions

**Figure 8:**
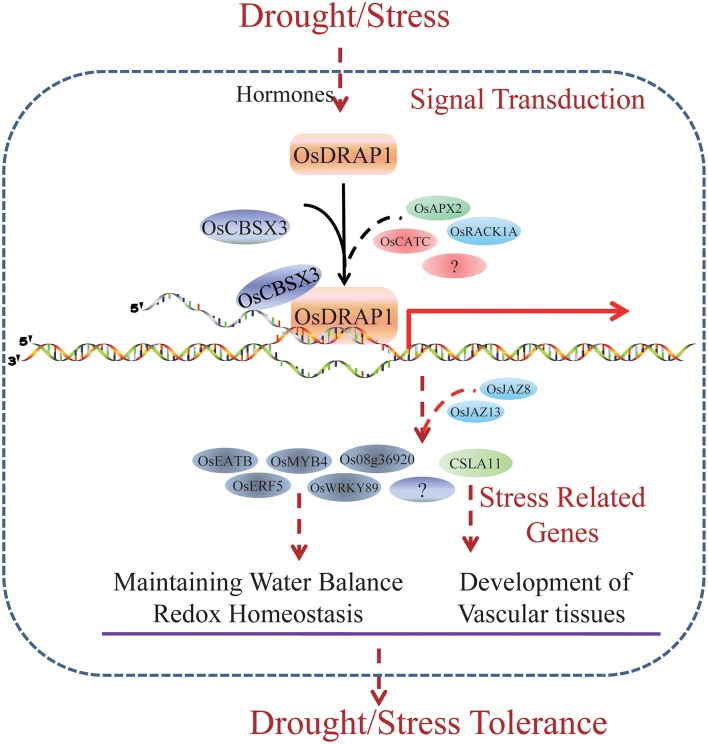
A model for *OsDRAP1*-Mediated Stress Tolerance. Through a set of plant hormones pathways, the rice feels the stress signal and induce the expression of *OsDRAP1*. Interacting with other stress related proteins, including OsCBSX3, OsRACK1A, OsRZFP34, OsAPX2, and OsCATC, OsDRAP1 activate stress responsive genes which regulate water balance, redox homeostasis and vascular development directly or indirectly. Several stress responsive TFs (*OsEATB, OsERF5, OsMYB4*, and *OsWRKY89*) and a gene encoding cellulose synthase-like family A (CSLA11) induced by OsDRAP1 complex are likely to mediated by JA signaling pathway (*OsJAZ8, OsJAZ13, Jasmonate-zim-domain protein 1*, and *Jasmonate O-methyltransferase*). *OsDRAP1* mediate stress tolerance especially DT via its regulation of these stress responding genes.

*OsDRAP1* is a DREB2-like TF gene which affects DT by maintaining water balance and redox homeostasis in rice under water deficit conditions. *OsDRAP1* appears to be involved in the regulation of the development of the vascular tissues. Constitutive overexpression of *OsDRAP1* could improve drought stress tolerance by affecting downstream genes with GCC and DRE *cis*-elements and synergistically regulating several TF genes including *OsERF5, OsMYB4*, and *OsWRKY89* and those related to JA pathways (Figure 8). The interaction protein OsCBSX3 could participate in the OsDRAP1-mediated regulation network in drought stress tolerance in rice.

## Author contributions

LH and YW generated the transgenic materials, designed and performed the experiments, WW and ZcL analyzed RNA-Seq data, FS and QQ provided assistance on vector construction, XZ, FH, and YZ provided assistance on the drought phenotyping experiment, BF and ZL designed the experiments and wrote the manuscript.

### Conflict of interest statement

The authors declare that the research was conducted in the absence of any commercial or financial relationships that could be construed as a potential conflict of interest.

## Supplementary material

Figure S1 The phylogenetic analysis of the DREB proteins in plants. The maximum parsimony tree of the DREB proteins from various plants **(A)** and rice **(B)**, based on the sequence database in UniProt Knowledgebase (UniProtKB): *Arabidopsis thaliana, At; Oryza sativa, Os; Zea mays, Zm; Phyllostachysedulis, Pe; Helianthus annuus, Ha; Oryzabrachyantha, Ob; Aegilopsbiuncialis, Ab; Nicotianabenthamiana, Nb; Poapratensis, Pp; Triticumaestivum, Ta; Glycine max, Gm; Solanumlycopersicum, Sl; Festucaarundinacea, Fa*.

Figure S2 Expression analysis of *OsDRAP1* in the transgenic rice plants. With red and green columns indicating *OsDRAP1* overexpression (OsDRAP1-OE-1~13) and *OsDRAP1* RNAi (OsDRAP1-RNAi-1~4) in transgenic lines, respectively, the error bars indicating SD based on data of 3 replicates, *Actin 1* used as the endogenous control and Arrows indicating the two lines (OE-7 and RNAi-2) that were used for further functional analysis.

Figure S3 The negative effect on the plants overexpressing and knock-down of *OsDRAP1*. **(A)** The growth of *OsDRAP1*-OE-7, WT and *OsDRAP1*-RNAi-2 plants. Investigation of plant height **(B)**, number of tillering **(C)**, root performance **(D)**, fertility **(E)**, and grain yield **(F)** of the transgenic lines. The asterisk indicated the significant difference in *t*-test (*p* < 0.05 vs. WT, *n* > 10).

Table S1 List of the differentially expressed gene in the *OsDRAP1* overexpression line compared with WT under normal growth conditions.

Table S2 List of the differentially expressed gene in *OsDRAP1* overexpression line compared with WT under drought stress conditions.

Table S3 List of interacting proteins of OsDRAP1 identified by yeast two-hybridization screening.
