# Supplementary figures and images for: Characterization of Transcription Factor Gene OsDRAP1 Conferring Drought Tolerance in Rice
Source: Front Plant Sci. 2018 Feb 1;9:94. doi: 10.3389/fpls.2018.00094 (PMC5799227; doi:10.3389/fpls.2018.00094)

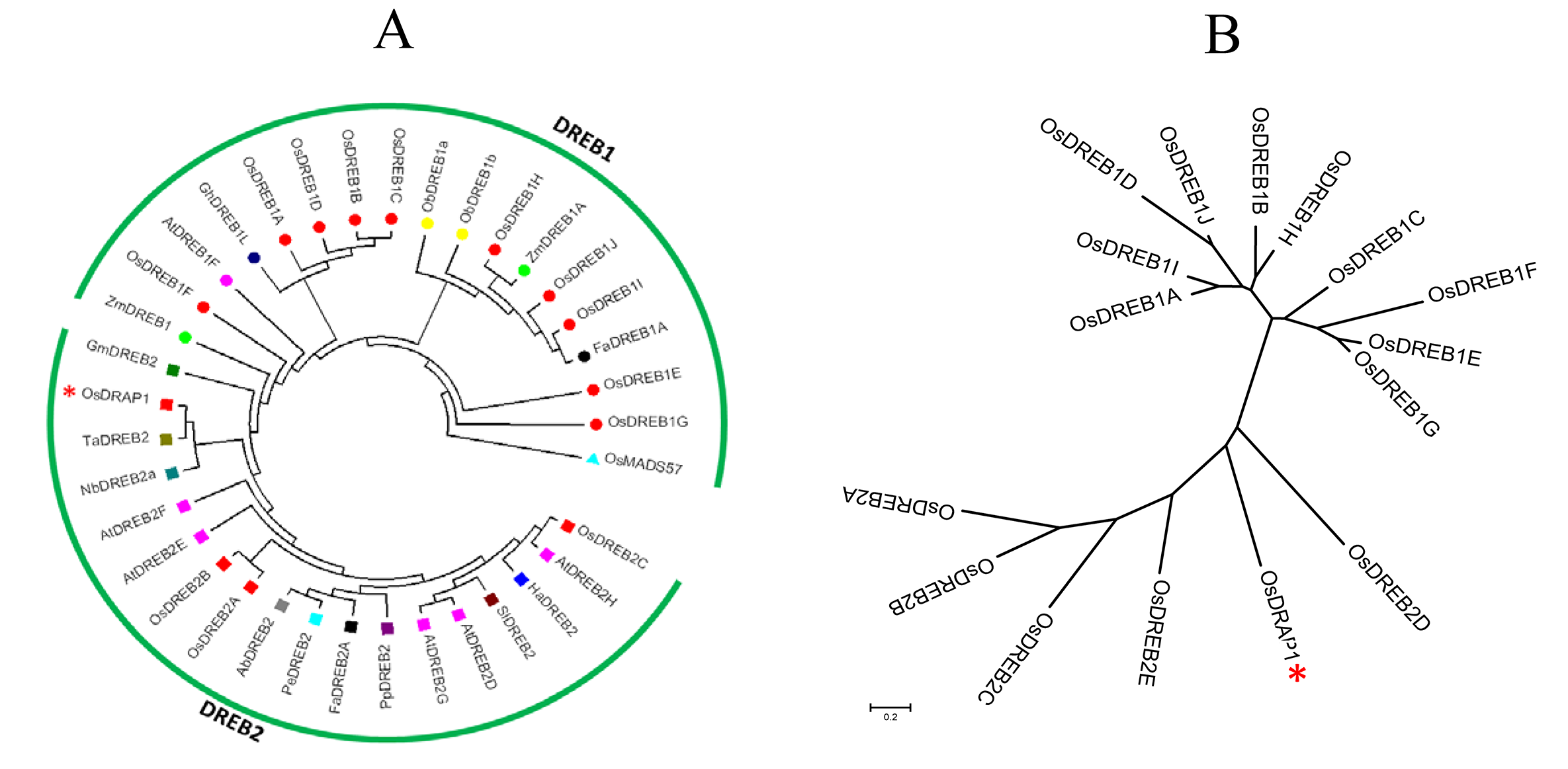

Supplement: Figure S1 — The phylogenetic analysis of the DREB proteins in plants. The maximum parsimony tree of the DREB proteins from various plants (A) and rice (B), based on the sequence database in UniProt Knowledgebase (UniProtKB): Arabidopsis thaliana, At; Oryza sativa, Os; Zea mays, Zm; Phyllostachysedulis, Pe; Helianthus annuus, Ha; Oryzabrachyantha, Ob; Aegilopsbiuncialis, Ab; Nicotianabenthamiana, Nb; Poapratensis, Pp; Triticumaestivum, Ta; Glycine max, Gm; Solanumlycopersicum, Sl; Festucaarundinacea, Fa. [file Image1.TIF]

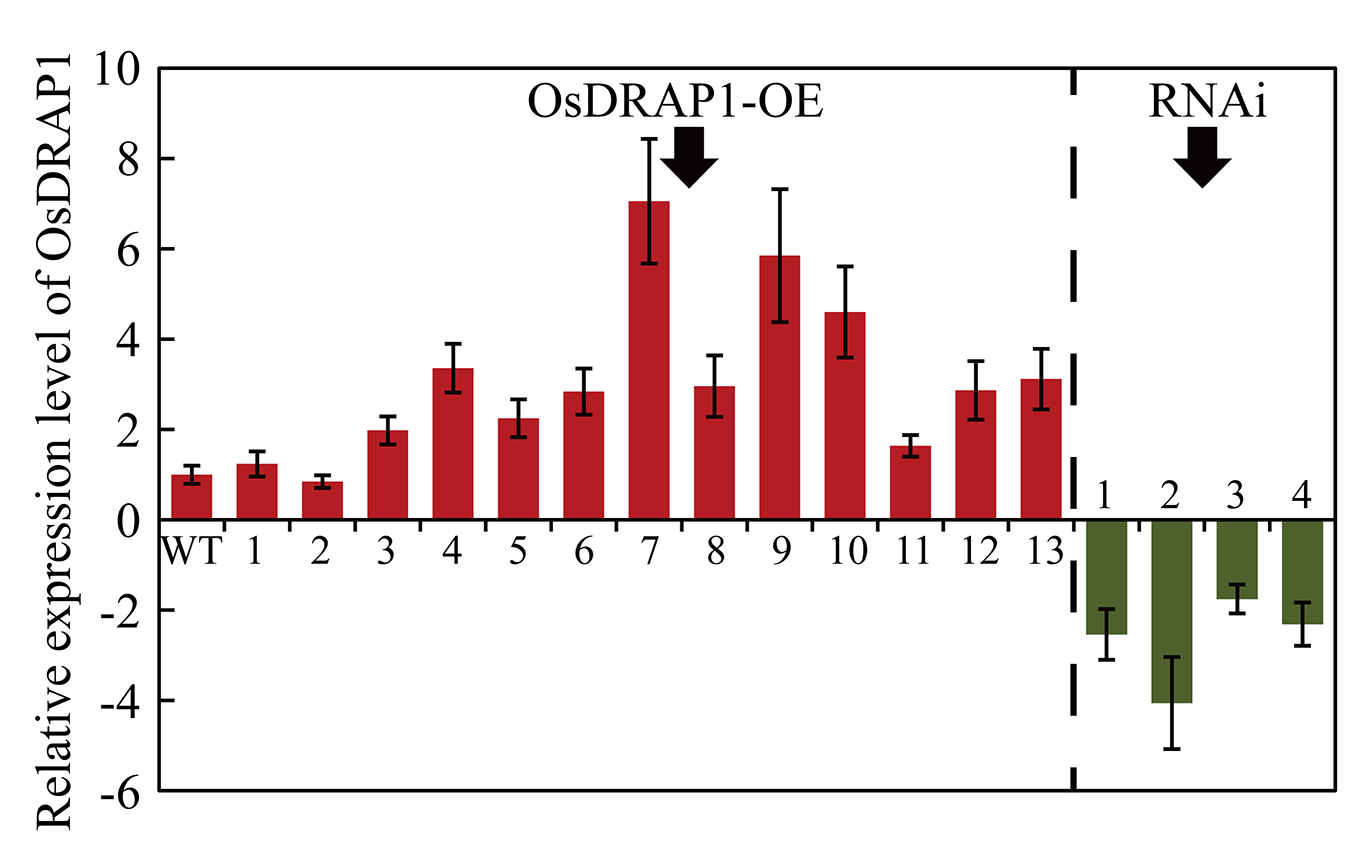

Supplement: Figure S2 — Expression analysis of OsDRAP1 in the transgenic rice plants. With red and green columns indicating OsDRAP1 overexpression (OsDRAP1-OE-1~13) and OsDRAP1 RNAi (OsDRAP1-RNAi-1~4) in transgenic lines, respectively, the error bars indicating SD based on data of 3 replicates, Actin 1 used as the endogenous control and Arrows indicating the two lines (OE-7 and RNAi-2) that were used for further functional analysis. [file Image2.TIF]

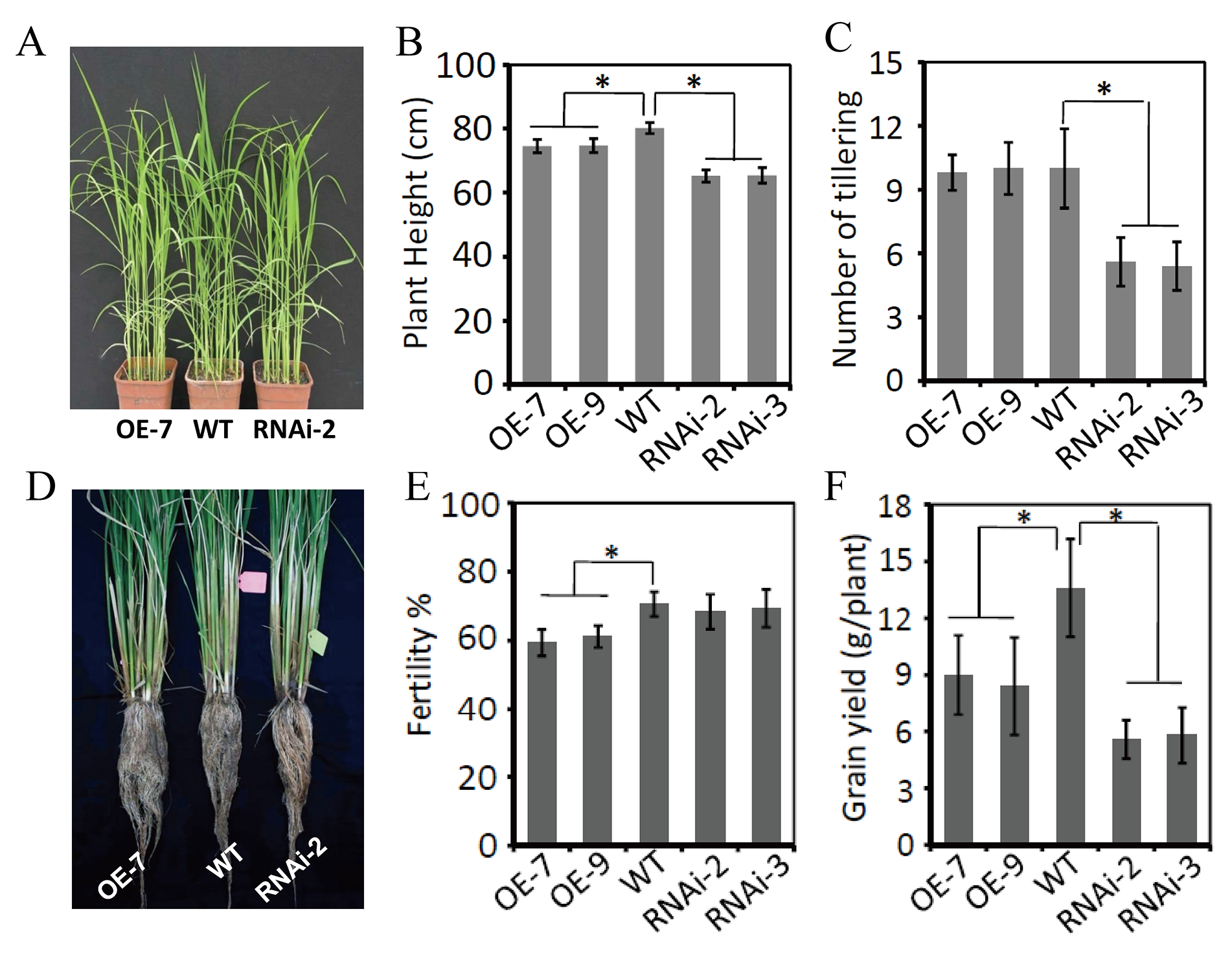

Supplement: Figure S3 — The negative effect on the plants overexpressing and knock-down of OsDRAP1. (A) The growth of OsDRAP1-OE-7, WT and OsDRAP1-RNAi-2 plants. Investigation of plant height (B), number of tillering (C), root performance (D), fertility (E), and grain yield (F) of the transgenic lines. The asterisk indicated the significant difference in t-test (p < 0.05 vs. WT, n > 10). [file Image3.TIF]
